# Precision medicine: an intrahepatic cholangiocarcinoma with a novel RBPMS-MET fusion sensitive to crizotinib

**DOI:** 10.1093/oncolo/oyae340

**Published:** 2024-12-10

**Authors:** Wei Wan, Xueqin Liu, Yamin Zhang, Rui Shen, Weihu Xia, Shuangni Li, Yuan Tan, Qianqian Duan, Jinpeng Liu, Wuping Wang

**Affiliations:** Department of Medical Oncology, Xi’an International Medical Center, Shaanxi, Xi’an, 710100, People’s Republic of China; Department of Medical Oncology, Xi’an International Medical Center, Shaanxi, Xi’an, 710100, People’s Republic of China; Department of Medical Oncology, Xi’an International Medical Center, Shaanxi, Xi’an, 710100, People’s Republic of China; Department of Medical Oncology, Xi’an International Medical Center, Shaanxi, Xi’an, 710100, People’s Republic of China; Department of Medical Oncology, Xi’an International Medical Center, Shaanxi, Xi’an, 710100, People’s Republic of China; Department of Gastroenterology, Xi’an International Medical Center, Shaanxi, Xi’an, 710100, People’s Republic of China; The State Key Lab of Translational Medicine and Innovative Drug Development, Jiangsu Simcere Diagnostics Co., Ltd, Nanjing, 210018, People’s Republic of China; The State Key Lab of Translational Medicine and Innovative Drug Development, Jiangsu Simcere Diagnostics Co., Ltd, Nanjing, 210018, People’s Republic of China; Department of Medical Oncology, Xi’an International Medical Center, Shaanxi, Xi’an, 710100, People’s Republic of China; Department of Thoracic Surgery, Xi’an International Medical Center, Shaanxi, Xi’an, 710100, People’s Republic of China

**Keywords:** ICC, NGS, RBPMS-MET fusion, crizotinib

## Abstract

**Background:**

Intrahepatic cholangiocarcinoma is a malignant tumor that starts from the epithelium of the bile duct and has a poor prognosis. They are characterized by poor response to chemotherapy and lack of effective targeted therapies; thus, therapeutic options are limited.

**Case Presentation:**

A 59-year-old man was admitted to the hospital for a workup of abnormal CA19-9 levels. He was diagnosed with ICC, underwent surgery and was found to have pT1bNx disease. He developed rapid disease recurrence on adjuvant gemcitabine + capecitabine. Following recurrence, he received first-line systemic pembrolizumab + lenvatinib and second-line pembrolizumab + lenvatinib + chemotherapy and had mild tumor regression followed by progression. Next-generation sequencing was performed on the baseline surgical sample. This revealed a novel RBPMS-MET fusion, and based on the literature, crizotinib 250 mg twice a day was administered. After 3 months of crizotinib treatment, magnetic resonance imaging revealed a significant reduction in liver lesions, and 4 months after initiating treatment, scans demonstrated a partial response.

**Conclusion:**

Our case report strengthens the evidence that crizotinib may be a viable treatment option for patients with ICC with a c-MET tyrosine kinase fusion, necessitating additional clinical investigation.

**Key points**

- To the authors’ knowledge, this is the first reported case of RBPMS‐*MET* fusion in ICC.
- *MET* fusion is rare in ICC, and MET tyrosine kinase inhibitors could be a option for ICC that warrants further clinical investigation.

## Introduction

Intrahepatic cholangiocarcinoma (ICC) is the second most prevalent primary liver cancer, accounting for up to 20% of all hepatic malignancies and 3% of gastrointestinal malignancies.^1^ Complete surgical resection remains the only potential cure for ICC, but only 20%-30% of patients present with resectable disease. Even after curative‐intent surgical resection, the 5‐year OS rate is approximately 20%-35%.^2^ Recent comprehensive genetic analysis has better characterized the genomic and transcriptomic landscape of cholangiocarcinoma subtypes classified as intrahepatic or extrahepatic.^3^ Promising candidates for targeted, personalized therapy have emerged, including potential driver fibroblast growth factor receptor gene fusions.^4^ Dysregulation of the c-MET tyrosine kinase (MET) is an established driver of oncogenesis. MET is unique in that 3 different genomic states can lead to clinically relevant oncogenesis: amplification, mutation, and fusion.^5^ A recent case report described a patient with ICC harboring a MET fusion who achieved a response to crizotinib.^6^ In this case report, we highlight a patient with ICC harboring a novel RBPMS-MET fusion who experienced a dramatic response to crizotinib.

## Case presentation

**Figure 1.**
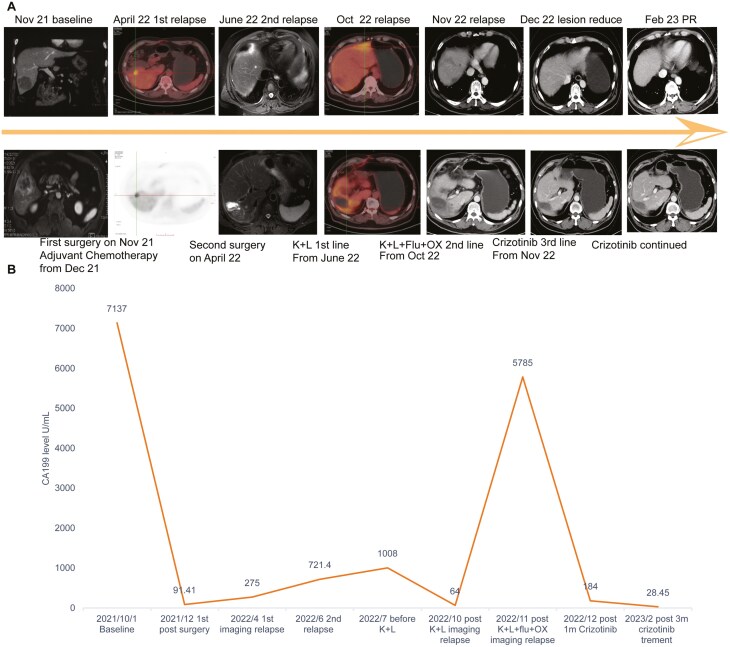
(A) Treatment timeline and representative imaging examinations. (B) Dynamic changes of CA19-9 with treatment. Note: K+L: pembrolizumab Lenvatinib; K+L+Flu+OX: pembrolizumab Lenvatinib+ fluorouracil+ oxaliplatin.

**Figure 2.**
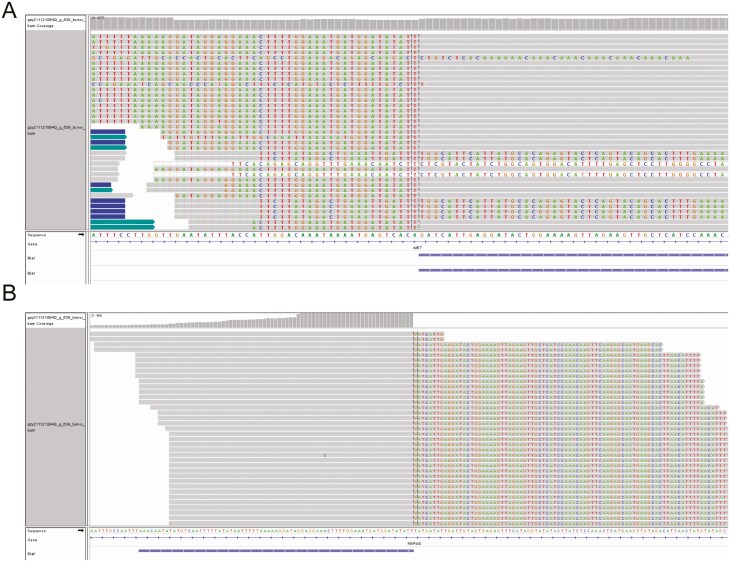
Integrative genomics viewer (IGV) image of RBPMS-MET fusion of this patient. (A) IGV image of MET part; (B) RBPMS part of IGV.

A 59-year-old man presented in October 2021 for a workup of elevated CA 19-9 (7137 U/mL). Liver-enhanced magnetic resonance imaging (MRI) revealed a 4.1 × 4.5 × 6.2 cm lesion in the posterior lower segment of the right lobe of the liver. Enhanced computerized tomography (CT) showed a 5.0 × 4.7 cm hypodensity in the same location (Figure 1A). He underwent laparoscopic partial hepatectomy and regional lymphadenectomy of the porta hepatis with negative margins. The patient had ICC with a staging of pT1bNx. Moreover, a 639 cancer-related gene next-generation sequencing (NGS) panel analysis was performed on the surgical sample by a College of American Pathologists accredited lab in November 2021. No targetable alterations were detected, but a novel RBPMS-MET fusion was identified. Tumor mutation burden was 5.7/Mb, and the tumor was microsatellite stable. The patient’s CA 19-9 level decreased to 91.41 U/mL after surgery (Figure 1B). Four cycles of chemotherapy (gemcitabine intravenous 1000 mg/m^2^ for 30 minutes on days 1 and 8; and capecitabine 1250 mg/m^2^ twice daily every 3 weeks from day 1 to day 14) were administered beginning December 2021. Then, CA19-9 rose to 275 U/mL, and in April 2022, PET/CT revealed a 1.95 cm nodular hypodensity near the liver with increased SUVmax uptake of 13.97 (Figure 1A); local recurrence was evaluated. The patient subsequently underwent a second partial hepatectomy with negative margins. However, CA 19-9 rose to 1008.00 U/mL after the second surgery, and there was an abnormal signal in the liver on MRI (Figure 1A). Then, the patient was treated with pembrolizumab 200 mg (on cycle 1 day 1) combined with lenvatinib 12 mg orally once daily. There was an initial decrease in CA 19-9 but after 4 months of treatment with pembrolizumab + lenvatinib, the CA 19-9 continued to rise. A CT scan revealed progressive disease in the liver (Figure 1A), and the patient was treated with pembrolizumab 200 mg (on cycle 1 day 1), lenvatinib 8 mg orally once daily, and oxaliplatin 85mg/m^2^ and fluorouracil 500 mg every 3 weeks, for a total of 2 cycles,^7,8^but the CA19-9 rose to 5785 U/mL, and an abdominal CT scan detected new lesions in bilateral anterior lobes of the liver, measuring right dominant lesion rose to 5.6 × 3.3 cm (Figure 1A and B). Patients with lung adenocarcinoma patients and ICC harboring EHBP1 or KIF5B or CAV1-MET fusions have achieved responses to crizotinib in previous case reports^6,9,10^ (Figure 2). The patient next began crizotinib 250 mg per day in November 2022 in view of the novel RBPMS-MET fusion. After 4 months of crizotinib therapy, the CA19-9 level downtrended from 5785U/mL to 28.45 U/mL (Figure 1B). The dominant liver lesion decreased (from 5.6 × 3.3 cm to 3.8 × 1.8 cm), along with other sites. The patient’s clinical assessment revealed a partial response (PR) based on RECIST v1.1 criteria. The patient has maintained a PR while continuing to take 250 mg of crizotinib twice daily up until July 2023. Other than mild bilateral edema of the lower limbs and hypoproteinemia (minimum albumin level of 31g/L), no additional adverse events associated with crizotinib were identified.

## Discussion

The dysregulation of the receptor tyrosine kinase c-MET (henceforth MET) is a well-established oncogenic driver. MET can be distinguished from numerous other proto-oncogenes by permitting 3 distinct types of genomic alterations to induce oncogenesis that are clinically significant: fusion, mutation, and amplification.^5^ In contrast to MET mutations and amplification, MET fusions are less common, but have been identified in multiple tumor types including lung cancer and glioblastoma.^11,12^ Crizotinib is a small-molecule tyrosine kinase inhibitor that inhibits MET, anaplastic lymphoma kinase (ALK), and C-ros oncogene 1, receptor tyrosine kinase (ROS1). The Food and Drug Administration of the United States has approved crizotinib for advanced non-small cell lung cancer with ALK or ROS1 fusion.^13^ Response to crizotinib has been documented in case reports of patients with lung adenocarcinoma with MET fusion. For instance, patients with lung cancer harboring the KIF5B‐MET fusion had responses of 8-10 months’ duration to crizotinib.^11^ A PR was noted in a 62‐year‐old woman with lung cancer harboring a STARD3NL‐MET gene fusion(69% reduction).^14^ ICC rarely exhibits MET fusions. A retrospective study conducted by Xu et al^15^ revealed that MET rearrangement was seen in only 1.1% of patients with biliary tract cancer (BTC). To date, only 2 case reports have documented instances of patients with ICCs harboring MET fusions (specifically EHBP1‐MET or CAPZA-2-MET) that were sensitive to MET inhibitors crizotinib or capmatinib, respectively.^6,16^ In this case report, we describe a patient with ICC harboring a novel RBPMS-MET fusion who experienced a remarkable response to crizotinib. This finding, alongside previously reported cases of MET fusion in ICC patients, further supports the notion that crizotinib may represent an effective treatment option for patients with ICC that harbor MET fusions.

Ongoing clinical trials are evaluating the efficacy of MET tyrosine kinase inhibitors in various solid tumor types to determine whether MET alterations can be considered a tumor-agnostic target (NCT02978261, NCT03993873, and NCT01639508). MET alterations in the context of ICC warrant further investigation, and as noted above, it is important to recognize gene fusions as a target for precision medicine (REFERENCE). Future research must investigate the integrated kinase fusion landscape of ICC and their sensitivity to TKI therapy.

## Data Availability

All data generated or analyzed during this study are included in this article.
